# A Mini Review Validating the Therapeutic Potential of *Psorospermum febrifugum* Spach Extracts in the Treatment of Acne Vulgaris

**DOI:** 10.1155/tswj/4016492

**Published:** 2025-10-02

**Authors:** Simon Peter Kaweesa, Tony Wotoyitide Lukwago, Martin Odoki, Ibrahim Ntulume, Godwin Anywar, Washington Willy Anokbonggo, Bernard Guyah, James Ombaka

**Affiliations:** ^1^Department of Biomedical Science and Technology, School of Public Health and Community Development, Maseno University, Maseno, Kenya; ^2^Department of Pharmacology & Therapeutics, School of Medicine, Health & Life Sciences, King Ceasor University, Kampala, Uganda; ^3^Department of Pharmacology & Toxicology, School of Pharmacy, Kampala International University Western Campus, Bushenyi, Uganda; ^4^Department of Microbiology and Immunology, School of Medicine, Health & Life Sciences, King Ceasor University, Kampala, Uganda; ^5^Department of Applied Sciences, School of Sciences, Nkumba University, Entebbe, Uganda; ^6^Ethnopharmacology and Zoopharmacognosy, Bernhard Nocht Institute for Tropical Medicine, Hamburg, Germany; ^7^Department of Plant Science, Microbiology and Biotechnology, College of Natural Sciences, Makerere University Kampala, Kampala, Uganda

**Keywords:** acne vulgaris disease, efficacy, formulation potential, garcinone E, lipase inhibition, oxidative stress, prenylated xanthones, *Psorospermum febrifugum*, safety

## Abstract

**Background:**

This study explored the potential of *Psorospermum febrifugum* extracts as a promising natural treatment option for acne vulgaris. Previous research suggests that *P. febrifugum* extracts might hold promise against factors that worsen acne vulgaris. This valuable information is however still scattered across various scientific publications, and this has hindered its accessibility, comprehensive understanding, and easy usage. There is need to review, synthesize, and compile this valuable information into a user-friendly article to enable easy understanding, accessibility, and usability of *P. febrifugum* bark extract as an option to treat acne vulgaris.

**Methods:**

A comprehensive assessment, synthesis, and compilation of the potential of *P. febrifugum* for treatment of acne vulgaris were conducted through review of work done across major scientific databases (Cochrane Library, MEDLINE/PubMed, Web of Science, Scopus, and Google Scholar), among studies published in English between April 1980 and April 2022. The search focused on studies that investigated the plant description, traditional medicinal usage, active components of *P. febrifugum*, its safety profile, and its efficacy against factors contributing to acne vulgaris lesions. The study included *in vitro*, *in vivo*, *ex vivo*, and clinical trials while excluding computer-modeled (*in silico*) studies.

**Results:**

From an initial 200 search results, 37 full-text articles inclusive of three informative abstracts met the inclusion criteria. The review revealed that *P. febrifugum* extracts possessed several properties beneficial for acne treatment, including anti-inflammatory response, antibacterial properties, antioxidant activity, antilipase activity, and keratolytic properties (promoting skin cell shedding). Notably, key bioactive compounds such as garcinone E, psorospermin, mangiferin, and betulinic acid exhibit distinct structural features, including prenylation and hydroxylation, that contribute to their ability to inhibit microbial growth, suppress inflammation, regulate lipid metabolism, and all critical pathways in acne pathogenesis. These structural–functional relationships between phytochemicals and acne mechanisms support the pharmacological promise of this plant extract.

**Conclusion:**

The review suggests that *P. febrifugum* holds significant therapeutic potential against factors that aggravate acne vulgaris severity. The stem bark of *P. febrifugum* is therefore a promising raw material that could be used to develop topical formulations (gels or creams) for the treatment of acne vulgaris disease.

## 1. Introduction

Acne vulgaris is a common skin condition affecting nearly 650 million people globally [1]. It currently has significant psychological consequences for adolescents, leading to feelings of humiliation, depression, and social withdrawal [2]. While conventional treatments like salicylic acid, adapalene, and retinoids are available, they may not be effective for all types of acne vulgaris, particularly those involving bacterial infections [3, 4]. Additionally, traditional antibiotics that are being used against acne-causing bacteria (*Propionibacterium acnes* and *Staphylococcus epidermidis*) are facing increasing resistance [5]. Currently, benzoyl peroxide remains an option to which resistance has not been developed, but its potential for skin irritation, bleaching, and burning sensations limits its use [6]. This therefore reveals the urgent need to search for effective and well-tolerated drug options to treat acne vulgaris.

Since time immemorial, medicinal plants have served as important therapeutic agents. Currently, they continue to provide new promising substitutes for antiacne treatments with distinctive mechanisms of antimicrobial activities, as reviewed by Cristani and Micale [7]. A growing body of contemporary research now supports the therapeutic relevance of plant-based agents in targeting multiple pathogenic factors of acne vulgaris. A review by Ajao and Moteetee [8] demonstrated the superiority of the phytochemicals that offer antimicrobial, anti-inflammatory, antioxidant, and sebum-regulatory activity interventions and are not adequately addressed by conventional monotherapies. One of such plants that has of recent provided promising unique multiple therapeutic action against acne vulgaris is *Psorospermum febrifugum* Spach; it is a 4–6 m tall shrub or small tree in the Hypericaceae family with a bumpy stem and a long history of use in traditional medicine for treating several skin ailments [8]. Traditionally, it is being used to treat acne vulgaris, subcutaneous wounds, leprosy [9], parasitic diseases (craw-craw and scabies), eczema, insect bites, epilepsy, and herpes zoster [10].

Extracts from *P. febrifugum* have recently demonstrated potential efficacy against several factors contributing to acne vulgaris development [11, 12]. This suggests that *P. febrifugum* is a promising natural drug candidate that could be used to develop safe and effective treatment options for acne vulgaris. *P. febrifugum* extracts are exceptionally known to target four key mechanistic factors involved in the pathological aggravation of acne vulgaris, and these include (1) inhibition of acne-causing bacteria: a study by Ajao and Moteetee [8] demonstrated that *P. febrifugum* extracts from both the stem bark and leaves significantly inhibited *P. acnes* colonization, which is responsible for the pathogenicity of acne vulgaris. (2) Lipase enzyme inhibition: research by Ajao and Moteetee [8] demonstrated that *P. febrifugum* extracts inhibited lipase, an enzyme that breaks down triglycerides in sebum into free fatty acids. Free fatty acids can irritate the skin and contribute to the formation of comedones, a hallmark of acne vulgaris. (3) Antioxidant activity: *P. febrifugum* extracts possess strong antioxidant properties [13]. These properties may help reduce inflammation and oxidative stress in the skin, both of which can worsen acne vulgaris [12]. (4) Anti-inflammatory effects: *P. febrifugum* extracts may have anti-inflammatory properties that could help mitigate inflammation associated with acne lesions [11].

Despite this promise, the valuable information regarding the diversity of bioactive compounds (Table 1) and the therapeutic potential of *P. febrifugum* extracts for acne vulgaris treatment is still fragmented across existing scientific publications, hindering accessibility, comprehensive understanding, and broader application. Moreover, a deeper exploration of its bioactive components in Table 2, most especially the prenylated xanthones like garcinone E and psorospermin, reveals structural traits such as hydroxylation and isoprenyl side chains that facilitate both dermal penetration and biochemical interaction with inflammatory and microbial targets [16]. The current study therefore reviewed, synthesized, and collated this information to enable greater understanding, accessibility, and usability. This synthesis is particularly timely given the emergence of formulation science focused on multitargeted, plant-derived topical therapies that are safer and more justifiable than traditional acne treatments. This undertaking is aimed at supporting both the clinical use and innovations of *P. febrifugum*–based treatment options for acne vulgaris.

## 2. Methods

### 2.1. Literature Search

To comprehensively assess the potential of *P. febrifugum* for acne vulgaris treatment, a mini review was conducted. A search strategy was developed using keywords related to *P. febrifugum*, acne vulgaris, and relevant mechanisms of action. This search was executed across major scientific databases (Cochrane Library, MEDLINE/PubMed, Web of Science, Scopus, and Google Scholar). The retrieved studies were thereafter subjected to a two-stage screening process. First, titles and abstracts were screened to identify potentially relevant articles based on predefined inclusion and exclusion criteria. These criteria included the following:

### 2.2. Inclusion Criteria

Studies published in English between April 1980 and April 2022 that focused their investigations on factors contributing to acne vulgaris development, safety profile, or efficacy of *P. febrifugum* extracts and its bioactive compounds. Still, *in vitro*, *in vivo*, *ex vivo*, and clinical trial research on the management of acne vulgaris were included.

### 2.3. Exclusion Criteria

The computer-modeled (*in silico*) studies were excluded from the review list.

### 2.4. Data Analysis

Full-text articles of potentially relevant studies were retrieved and reviewed. Data extraction was performed using a standardized data collection form designed to capture key information from each study, including study design, methodology, *P. febrifugum* preparation, investigated mechanisms of action, efficacy outcomes, and safety data. The extracted data was then subjected to a qualitative analysis. Studies were categorized based on their research focus (active components, safety, or efficacy) and mechanisms of action investigated. A narrative synthesis was conducted to summarize the findings from the included studies, identifying key themes and potential areas of future research.

### 2.5. Results of the Study

The initial search across various databases yielded 200 articles. After applying the inclusion and exclusion criteria, the review included 37 full-text articles from original research, inclusive of three informative abstracts (written in English but with non-English full texts).

#### 2.5.1. Description of *P. febrifugum*

*P. febrifugum* is a flowering shrub or small tree in the Hypericaceae family, typically reaching heights of 4–6 m. It is easily recognized by its rough, bumpy stem and thrives in open woodlands, ranging from low- to mid-altitude ecological zones. Its small, inconspicuous flowers are creamy-white, fragrant, and covered in fine hairs, measuring approximately 8 mm in diameter. The fruit is a small, bright red berry, roughly 6 mm in diameter at maturity [8, 11].

#### 2.5.2. Traditional Medicinal Usage

*P. febrifugum* has a long-standing role in traditional medicine, especially in sub-Saharan Africa, where it is commonly used to treat skin and infectious diseases. Communities in Nigeria traditionally employ the stem bark and leaves to treat subcutaneous wounds, skin sores, and leprosy [15]. In Cameroon, the plant is used to manage bacterial skin infections and neurological conditions such as epilepsy [10]. In Central Uganda, the stem bark is widely used for treating parasitic skin diseases such as; "craw-craw" and scabies, as well as pimples, eczema, insect bites, and herpes zoster [9, 17].

In the Bukoba region of Tanzania, *P. febrifugum* stem bark is locally employed as a topical treatment for acne vulgaris, which is referred to in regional dialects simply as “pimples” [10, 15]. This widespread ethnomedicinal use supports the hypothesis that *P. febrifugum* possesses pharmacologically active compounds capable of addressing acne's multifactorial pathogenesis.

Notably, modern pharmacognostic analyses have now validated several of these traditional uses by confirming the presence of bioactive secondary metabolites (Tables 1 and 2) such as prenylated xanthones (e.g., garcinone E), flavonoids, alkaloids, and saponins that correspond to the antimicrobial, antioxidant, and anti-inflammatory activities observed in laboratory models (Tables 1 and 2).

### 2.6. Phytochemicals, Extraction, and Yields

Table 1 summarizes the major phytochemicals identified in *P. febrifugum* extracts derived from both the leaves and stem bark using various solvents. The qualitative abundance of these compounds varies with extraction technique, solvent polarity, and plant part used.

*P. febrifugum* is known to contain a wide range of phytochemical compounds with pharmacological relevance, extracted in varying concentrations depending on the method and solvent used. Extraction processes for the stem bark and leaves often rely on maceration, a technique involving soaking the plant material in a solvent at room temperature for at least 72 h with intermittent agitation. This method remains widely used due to its simplicity, low cost, and ability to preserve heat-sensitive compounds.

While maceration remains standard, additional methods such as infusion, percolation, and decoction are occasionally employed, though less frequently in pharmacological studies. Solvent choice was found to significantly impact yield and compound diversity. According to Chinweze et al. [13], dichloromethane (DCM) produced the highest extract yield (35.7%), followed by acetone (24.4%), methanol (22.7%), ethanol (6.25%), and hexane (2.3%). These solvent-dependent differences likely reflect the selective solubility of mid- to high-polarity compounds such as xanthones and flavonoids.

Among the phytochemicals identified, flavonoids and terpenoids consistently appeared in high abundance, particularly in stem bark extracts. These classes are known for their hydroxylation patterns and lipophilicity, which are critical to their antioxidant and anti-inflammatory activity. Alkaloids, glycosides, and saponins were moderately abundant, while tannins and anthraquinones were identified in lower quantities [11–13].

Importantly, several xanthone derivatives including simple xanthones and O- and C-prenylated xanthones such as garcinone E and psorospermin have been documented to exist primarily in the stem bark ethanolic extracts. These compounds are structurally characterized by a tricyclic aromatic xanthone core with lipophilic prenyl substitutions that enhance skin permeability and facilitate interaction with enzymes like lipase and inflammatory mediators such as COX-2 and NF-*κ*B (Tables 2 and 3). Their presence may account for the extract's ability to inhibit bacterial growth, lipase activity, and inflammatory cytokine production—three major pathogenic factors in acne vulgaris [20].

Terpenoids and steroids, also present in moderate to high concentrations, may contribute to keratolytic properties and the modulation of sebaceous gland function, aligning with observed therapeutic effects in acne management. Additionally, solvent-specific enrichment of phenolics in DCM and methanol fractions further supports their use in pharmacologically optimized extractions. These phenolic-rich extracts are associated with superior DPPH radical scavenging capacity and enhanced anti-inflammatory activity in vitro [7, 21].

Ultimately, the chemical complexity of *P. febrifugum* stem bark, combined with solvent-dependent extractability, provides a phytochemical platform rich in xanthones, flavonoids, and alkaloids, with structural features that align closely with therapeutic targets in acne vulgaris pathophysiology [13].

### 2.7. Pharmacological Relevance of *P. febrifugum* Extracts

The stem bark and leaves of *P. febrifugum* contain a complex array of phytochemicals (Tables 2 and 3), each contributing uniquely to its pharmacological potential in managing acne vulgaris. These compounds span xanthones, flavonoids, triterpenoids, anthraquinones, lignoids, and furanoxanthone hybrids, many of which exhibit multitarget activity relevant to acne pathogenesis [13].

The chemical diversity of *P. febrifugum* supports a broad range of antimicrobial effects against acne-causing bacteria (Table 4) and pharmacological activities directly targeting acne vulgaris pathogenesis (Tables 3 and 5). The prenylated xanthones, such as garcinone E and psorospermin, exhibit lipophilic characteristics, allowing them to penetrate biofilms, inhibit bacterial enzymes, and block inflammatory cascades including NF-*κ*B, COX-2, and iNOS [14, 22].

Flavonoids like quercetin and mangiferin possess hydroxyl-rich structures that permit antioxidant activity and modulate sebum-associated lipase activity (Table 6). These structures interact with inflammatory mediators and offer superior safety in long-term topical use compared to synthetic retinoids or benzoyl peroxide [21].

The triterpenoid betulinic acid exhibits powerful membrane-disrupting antibacterial activity and is notable for avoiding resistance due to its nonprotein-targeted mechanism. Its deep dermal penetration due to lipophilicity enables delivery to inflamed sebaceous units [23].

3-Geranyloxyemodin and emodin, both anthraquinones, are now recognized in *P. febrifugum* extracts. Their lipophilic geranyl side chains enhance skin permeability, while their anthraquinone cores are effective at suppressing nitric oxide (NO) and prostaglandin E2 (PGE), which are critical inflammatory mediators in acne lesions [14].

Isocadensin D, a unique xanthonolignoid, merges the pharmacophores of xanthones and lignans, promoting free radical scavenging and potential downregulation of inflammatory pathways like MAPK and NF-*κ*B. This makes it promising for persistent or cystic acne management, especially when combined with other flavonoids or phenolic acids [7, 24, 25].

Psorofebrin, belonging to the tetrahydrofurobenzofuranoxanthones, is a rare compound that integrates benzofuran and xanthone structures, enhancing multisite activity in skin inflammation and tissue repair. Its structure supports both receptor-mediated signaling modulation and antioxidant defense, making it well-suited for use in wound-healing gels or acne scar recovery treatments [26]. These phytochemicals act on multiple biochemical pathways central to acne vulgaris.

These structural properties of compounds such as prenylation, hydroxylation, glycosylation, and fused ring systems—not only enhance topical bioavailability but also enable formulation into cosmeceuticals like gels, creams, or patches targeting different stages of acne lesions.

### 2.8. Experimental Studies and Mechanistic Validation

Extracts from *P. febrifugum* have demonstrated notable bioactivity against multiple pathophysiological mechanisms that drive the development and persistence of acne vulgaris [11, 12, 15]. This evidence supports the consideration of *P. febrifugum* as a multitargeted, plant-based drug candidate with therapeutic potential for dermatological conditions. The bioactive phytochemicals within its extracts operate via four principal pathways relevant to acne vulgaris: inhibition of acne-causing bacteria, suppression of sebaceous lipase activity, antioxidant action, and modulation of inflammatory responses [8].

### 2.9. Inhibition of Acne-Causing Bacteria

*Cutibacterium acnes* (formerly *P. acnes*) and *S. epidermidis* are the principal microbial agents involved in acne vulgaris lesion formation. Experimental studies (Tables 3 and 4) have demonstrated that stem bark and leaf extracts of *P. febrifugum* inhibit the growth of these micro-organisms, with the stem bark extract displaying greater antibacterial activity [27]. The minimum inhibitory concentration (MIC) for the stem bark extract against *P. acnes* was 12.5 mg/mL, while that of leaf extract was 50 mg/mL [12].

The superior efficacy of the stem bark extract is attributed to its higher concentration of xanthones and phenolic compounds (Table 1), which disrupt bacterial cell membranes, interfere with protein synthesis, and inhibit respiratory enzymes. Notably, alkaloids present in both plant parts also contribute to bactericidal action, aligning with known antimicrobial mechanisms [12].

Beyond common acne pathogens, *P. febrifugum*'s phytochemical complexity includes unique antimicrobial agents such as febrifuquinone and adamabianthrone, which have shown broad-spectrum activity. These anthraquinone dimers, exclusive to the *Psorospermum* genus, are thought to interact with bacterial DNA via intercalation, while simultaneously exerting oxidative stress to inhibit growth [16].

Further, anthraquinones such as 3-geranyloxyemodin and emodin are reported to inhibit microbial growth and suppress inflammatory mediators (e.g., NO and PGE). The presence of geranyl side chains enhances dermal bioavailability, which is critical for the topical application of antiacne agents [7, 14].

#### 2.9.1. Anti-Inflammatory Properties

Chronic inflammation underlies many of the lesions in acne vulgaris (Table 5). Studies suggest that the ethanolic extract of *P. febrifugum* stem bark modulates the NF-*κ*B pathway, reducing the expression of COX-2 and iNOS, both of which are involved in proinflammatory mediator synthesis (PGE2 and NO) [12, 14].

In a carrageenan-induced paw edema model, oral administration of 400 mg/kg of the extract significantly inhibited edema formation, comparing favorably with indomethacin, a standard nonsteroidal anti-inflammatory drugs (NSAIDs) [11]. Another study using xylene-induced ear edema in mice confirmed the topical anti-inflammatory efficacy of the extract, comparable to dexamethasone, a corticosteroid [13]. Notably, compounds like garcinone E, betulinic acid, mangiferin, emodin, and psorofebrin have all been shown to influence inflammatory cascades, suppressing TNF-*α*, IL-6, and reactive oxygen species (Table 5).

### 2.10. Antioxidant Activity and Lipase Inhibition

Oxidative stress exacerbates acne by damaging skin lipids and proteins, especially after sebum oxidation by microbial lipase (Table 6). *In vitro* antioxidant testing (DPPH, TEAC, MCA, and FRAP) demonstrated that stem bark extracts had superior radical scavenging properties than both leaf extracts and vitamin C [13, 18].

Phenolic compounds, xanthones, and anthraquinones (e.g., isocadensin D, emodin, and psorofebrin) are key contributors to this antioxidant activity. These compounds scavenge reactive oxygen species, chelate pro-oxidant metals, and modulate endogenous enzymes like SOD and catalase [28, 29].

Additionally, lipase inhibition by *P. febrifugum* extracts is a critical antiacne mechanism, as lipase breaks down sebum into free fatty acids that induce inflammation and comedone formation. In one study, a 250 mg/mL methanolic extract of stem bark inhibited pancreatic lipase by 96%, compared to 82% inhibition by orlistat, a standard antiobesity drug used off-label in acne [12].

### 2.11. Mechanistic Implications of Experimental Findings

These experimental results reinforce the multitargeted therapeutic role of *P. febrifugum* extracts in acne vulgaris management contributed by the diverse phytochemicals (Table 7) as described by [8, 14, 27].

### 2.12. In Vivo Toxicity Studies

*In vivo* acute oral toxicity studies in rats and mice further reinforce these findings (Table 8). Doses as high as 5000 ppm and 5000 mg/kg, respectively, did not result in any mortality or observable signs of clinical toxicity [13, 14, 27, 30]. These findings confirm that even high oral doses of *P. febrifugum* ethanolic extracts are likely nonlethal and biocompatible under short-term exposure.

Additionally, dermal safety was assessed in rabbits, where application of aqueous stem bark extract at doses up to 10,000 mg/kg elicited no adverse effects on skin integrity, liver, or renal function [27]. This dermal safety finding is particularly relevant given the intended use of *P. febrifugum* in acne topical therapies.

Together, these findings validate the plant's suitability for safe dermal and oral administration at therapeutic doses, positioning it as a strong candidate for dermatopharmacological product development [8, 27, 30]. Still, long-term toxicity, reproductive toxicity, and allergenicity studies are essential for conclusive safety certification.

## 3. Discussion

This mini review provides a comprehensive pharmacological overview of *P. febrifugum*, synthesizing evidence that supports its therapeutic potential in treating acne vulgaris. The findings indicate that both stem bark and leaf extracts contain bioactive compounds that act on the multifactorial pathways involved in acne pathogenesis: microbial colonization, inflammation, oxidative stress, hyperkeratinization, and sebum dysregulation [8, 14].

The antibacterial action demonstrated by the stem bark extracts, particularly against *C. acnes* and *S. epidermidis*, confirms its efficacy in targeting one of the root causes of acne vulgaris [27]. This is attributed to the high concentration of structurally potent xanthones, such as garcinone E, and anthraquinones like 3-geranyloxyemodin and emodin [31–33]. These molecules are lipophilic and penetrate the skin efficiently, disrupting bacterial membranes and modulating bacterial DNA activity [33].

In addition to its antimicrobial action, *P. febrifugum* exhibits substantial antilipase activity, primarily attributed to flavonoids and xanthone derivatives such as mangiferin and quercetin. These compounds inhibit triglyceride breakdown within sebum, reducing the release of free fatty acids that aggravate comedone formation. This sebostatic effect is a critical advantage over conventional antibiotics, which often lack this mechanism [8, 12].

The review also underscores the antioxidant and anti-inflammatory capacity of this plant, both of which are important in countering acne-associated oxidative damage and immune-mediated responses. Compounds such as gallic acid, isocadensin D, and psorofebrin exhibit redox-scavenging and cytokine-suppressive properties. These phytochemicals target the NF-*κ*B pathway and inhibit key enzymes such as COX-2 and iNOS, known for promoting chronic skin inflammation [14, 34–37]. The extract's superiority over vitamin C in *in vitro* antioxidant assays supports its dermatological relevance [8].

Importantly, the inclusion of rare, structurally distinct compounds such as tetrahydrofurobenzofuranoxanthones (e.g., psorofebrin), xanthonolignoids (e.g., isocadensin D), and anthraquinone dimers (e.g., febrifuquinone and adamabianthrone) highlights the unique phytochemical profile of *P. febrifugum* [8]. These molecules are almost exclusive to the *Psorospermum* genus and demonstrate multiple, synergistic pharmacological activities that remain underexplored in mainstream acne therapy. Their hybrid molecular scaffolds support membrane interaction, enzyme binding, and receptor-mediated immunomodulation, offering promising alternatives to monotherapeutic agents such as clindamycin or retinoids [38].

Notably, the diverse phytochemical spectrum of *P. febrifugum* allows it to intervene across all major acne mechanistic axes—microbial, enzymatic, oxidative, and inflammatory—positioning it as a highly promising botanical candidate for further pharmaceutical development [8].

*P. febrifugum* has a well-documented history of use in traditional medicine across various regions, particularly in Africa, for dermatological and systemic ailments. This ethnomedical background supports its presumed safety profile [8, 39]. However, safety validation through preclinical models is essential for advancing toward clinical applications. Findings from cell-based and animal models are summarized in Table 8 and strongly suggest that *P. febrifugum* exhibits a favorable toxicity profile across multiple exposure routes and dose levels [30, 39].

*In vitro* cell toxicity studies using human glioblastoma cell lines have reported no observable cytotoxicity upon exposure to leaf and stem bark extracts, indicating good cellular tolerance [20]. This provides preliminary support for the extract's safety on human cells and warrants its exploration in topical applications. These results support further development of standardized extracts and formulations, although translational safety studies in humans are still required.

## 4. Conclusion

Multiple bioactive phytochemicals isolated from *P. febrifugum*, including prenylated xanthones (e.g., garcinone E), flavonoids (e.g., quercetin), anthraquinones (e.g., emodin), and triterpenoids (e.g., betulinic acid), exhibit mechanisms such as antimicrobial inhibition, lipase suppression, ROS scavenging, and inflammation modulation.

Notably, structurally unique phytochemicals such as febrifuquinone, adamabianthrone, psorofebrin, and isocadensin D—rarely found outside the *Psorospermum* genus—enhance the novelty and potential of the plant's pharmacological portfolio. These compounds engage with biochemical pathways integral to acne pathophysiology, such as COX-2/iNOS suppression and lipase-mediated sebum modulation.

Moreover, early safety studies indicate no significant toxicity up to high doses, affirming *P. febrifugum*'s favorable risk profile for dermatological development. However, comprehensive toxicological profiling, long-term exposure studies, and human trials remain essential.

## 5. Future Perspectives

To advance *P. febrifugum* from ethnobotanical use to pharmaceutical application, the following directions are proposed:

1. Standardized extraction and phytochemical characterization: Future studies must optimize solvent systems, quantify bioactive constituents, and validate compound stability for consistent batch-to-batch formulations.
2. Formulation innovation: The high lipophilicity of several xanthones and anthraquinones (e.g., emodin and garcinone E) supports the development of liposomal, gel-based, or nanoparticle-enhanced delivery systems for dermal penetration.
3. Clinical trials and human safety assessments: Controlled studies in human acne cohorts are urgently required to assess clinical efficacy, tolerability, and product usability.
4. Synergistic therapy development: Given its multitargeted profile, *P. febrifugum* extracts may serve as complementary agents alongside standard antiacne drugs (e.g., benzoyl peroxide and retinoids) to enhance outcomes and reduce resistance.
5. Cosmetic and cosmeceutical use: The antioxidant and skin-restoring properties of compounds like gallic acid and psorofebrin may benefit scar prevention and pigmentation control in postacne skincare formulations.

## Figures and Tables

**Table 1:** Phytochemical composition of stem bark and leaf extracts of *P. febrifugum* in different solvents.

**Extraction solvent/plant part**	**Identified phytochemicals**	**Relative abundance**	**Country**	**Reference(s)**
Xanthones (unspecified solvent)	Simple and prenylated xanthones	—	China	[17]
Ethanolic extract (stem bark)	Phenols, flavonoids, tannins, alkaloids, saponins, glycosides, steroids, terpenoids	+++	Nigeria	[13, 18]
Methanolic extract (leaves)	Phenols	+	Côte d'Ivoire	[19]
Ethanolic extract (stem bark), DCM fraction	Phenols, flavonoids, tannins, alkaloids, glycosides	++	Nigeria	[14]
Ethanolic extract (stem bark)	Alkaloids, flavonoids, steroids, tannins, terpenes, simple xanthones, O- and C-prenylated xanthones	+++	Nigeria	[15, 18]
Methanolic extract (leaves)	Alkaloids, tannins, saponins, cardiac glycosides, flavonoids	++	Nigeria	[12]
Methanolic extract (stem bark)	Alkaloids, tannins, saponins, cardiac glycosides, anthraquinones, flavonoids	+++	Nigeria	[12, 16]

*Note:* +++ = high abundance, ++ = moderate abundance, + = low abundance.

**Table 2:** Phytopharmacological relevance of compounds in *P. febrifugum* extracts against acne vulgaris.

**Compound**	**Structure type/feature**	**Key acne-relevant activities**	**I** **C**_50_ **/MIC values**	**Reference(s)**
Garcinone E	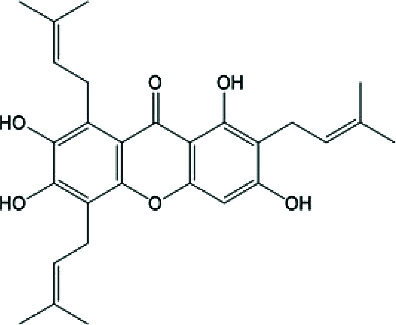 Prenylated polyhydroxylated xanthone	Antibacterial, antilipase, antioxidant, anti-inflammatory	MIC: 50–100 *μ*g/mL; DPPH: ~15–18 *μ*g/mL	[1, 2]
Mangiferin	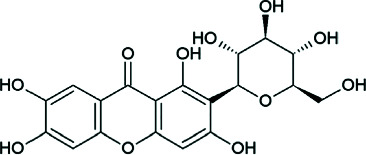 C-Glucosyl xanthone	Lipase inhibition, ROS scavenging, NF-*κ*B suppression	DPPH: 12.4 *μ*g/mL; lipase: 29.3 *μ*g/mL	[3, 4]
1,3,5-Trihydroxyxanthone	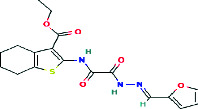 Simple hydroxylated xanthone scaffold	Mild antibacterial, antioxidant cofactor	—	[5]
Betulinic acid	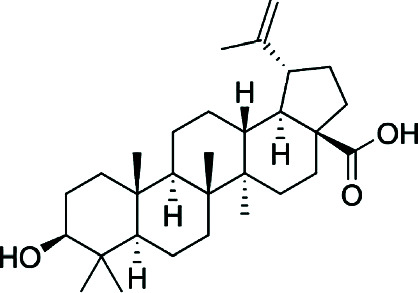 Pentacyclic triterpenoid with lipophilic lupane skeleton	Bacterial membrane disruption, COX-2/iNOS inhibition	MIC (*S. aureus*): ~125 *μ*g/mL	[6, 9]
Quercetin	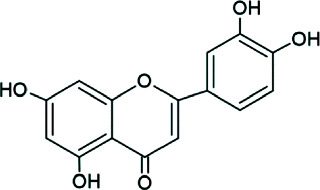 Flavonol with catechol B-ring	Lipase inhibition, cytokine suppression, antioxidant, desquamation control	Lipase: ~31.6 *μ*g/mL; DPPH: ~15.7 *μ*g/mL	[4, 7, 10]
Gallic acid	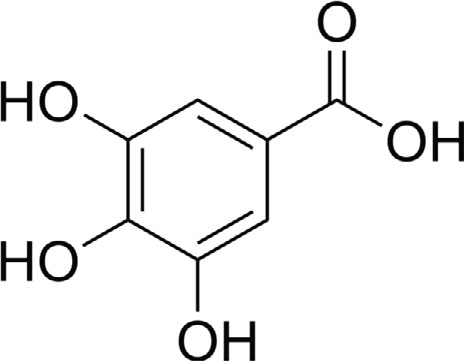 Trihydroxybenzoic acid	Potent antioxidant, mild antimicrobial, synergist in topical formulations	DPPH: 10.3 *μ*g/mL	[10]
3-Geranyloxyemodin	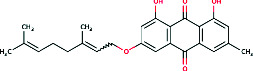 Lipophilic anthraquinone with geranyl side chain	Antimicrobial, NO and PGE2 inhibition, dermal penetration	—	[14]
Isocadensin D	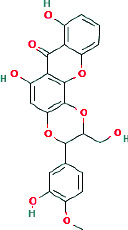 Xanthonolignoid (xanthone-lignan hybrid)	Antioxidant synergy, ROS modulation, cytokine suppression	—	[12]
Psorofebrin	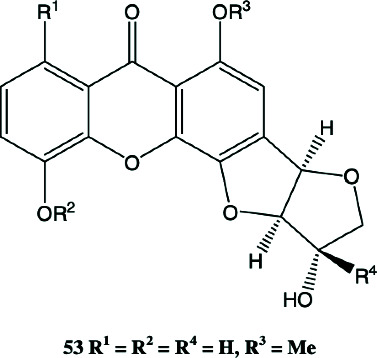 Tetrahydrofurobenzofuran-xanthone hybrid	Anti-inflammatory, wound healing, skin receptor interaction	—	[13]
Febrifuquinone	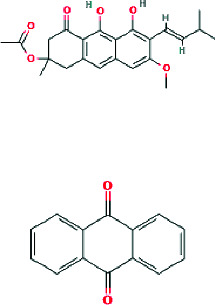 Vismione-anthraquinone coupled dimer	Broad-spectrum antimicrobial, DNA intercalation, oxidative stress response	—	[15]
Adamabianthrone	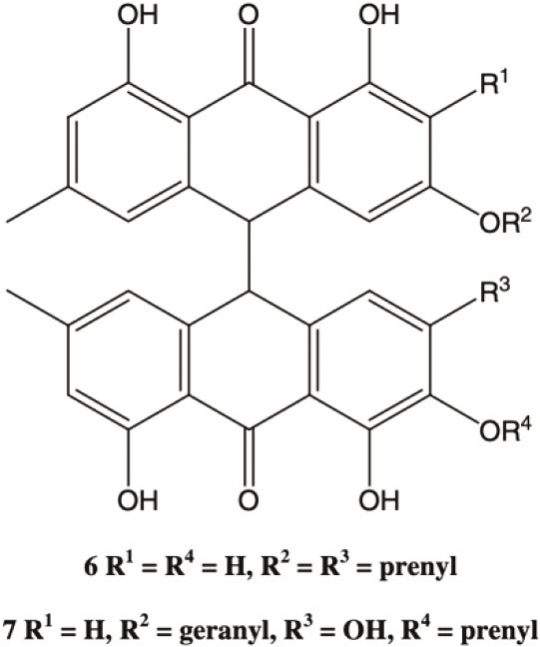 Anthraquinone dimer	Antibacterial, antifungal, oxidative damage induction in microbes	—	[15]

**Table 3:** Mechanistic effects of targeted compounds in *P. febrifugum* extracts.

**Pathogenic mechanism in acne**	**Targeted compounds in *P. febrifugum* extracts**
Bacterial growth and biofilm	Garcinone E, febrifuquinone, adamabianthrone, emodin
Lipase enzyme inhibition	Mangiferin, quercetin, garcinone E
ROS/oxidative stress	Gallic acid, isocadensin D, psorofebrin, mangiferin
Inflammatory cytokine inhibition	Betulinic acid, mangiferin, quercetin, emodin, psorofebrin
Sebum regulation	Betulinic acid, mangiferin, quercetin
Keratinocyte modulation	Quercetin, 1,3,5-trihydroxyxanthone
Lesion healing/tissue repair	Psorofebrin, isocadensin D, anthraquinones

**Table 4:** Inhibition of acne-causing bacteria by *P. febrifugum* extracts.

**Study/method**	**Intervention**	**Efficacy (*in vitro*)**	**Efficacy (*in vivo*)**	**Safety**	**Outcome**	**Reference(s)**
Agar diffusion method	Stem bark vs. leaf extract	MIC: 12.5 mg/mL (bark), 50 mg/mL (leaf) against *P. acnes*	Not applicable	Not explicitly stated	Inhibition attributed to phenolics, alkaloids, and xanthones	[11]
Microbial isolate testing	PFL, PFS, HPFS, EPFS, APFS (various solvent fractions)	Zones of inhibition and MICs provided across *S. epidermidis* and *P. acnes*	Not applicable	Not explicitly stated	Highest potency in PFS and EPFS fractions against both bacteria	[11]

Abbreviations: APFS, aqueous fraction; EPFS, ethyl acetate fraction; HPFS, *n*-hexane fraction; PFL, leaf extract; PFS, stem extract.

**Table 5:** Anti-inflammatory effects of *P. febrifugum* extracts.

**Study/method**	**Intervention/control**	**Efficacy (*in vivo*)**	**Outcome**	**Reference(s)**
Carrageenan-induced inflammation (rats)	400 mg/kg stem bark extract vs. indomethacin	Significant paw edema reduction at 1–3 h	Demonstrates systemic anti-inflammatory action	[13]
Xylene-induced ear edema (mice)	Topical extract vs. dexamethasone	Marked reduction in ear weight and inflammation	Supports topical efficacy for acne inflammation	[13]

**Table 6:** Antioxidant and lipase inhibition by *P. febrifugum* extracts.

**Therapeutic effect**	**Method/intervention**	***In vitro* efficacy**	***In vivo* efficacy**	**Safety**	**Outcome**	**Reference(s)**
Antioxidant properties	DPPH, TEAC, MCA, FRAP assays with leaf and bark extracts	Stem bark had higher scavenging activity	Not applicable	Not explicitly stated	Strong ROS neutralization linked to phenolics and xanthones	[11, 13]
Lipase inhibition	250 mg/mL methanolic bark extract	96% inhibition vs. 82% by orlistat	Not applicable	Not investigated	Supports sebum-regulating potential of extract	[12]

**Table 7:** Bioactive compounds from *P. febrifugum* and their targeted antiacne pathways.

**Acne pathway affected**	**Responsible compounds in *P. febrifugum***
Bacterial overgrowth	Garcinone E, febrifuquinone, adamabianthrone, emodin
Sebum breakdown (lipase activity)	Mangiferin, quercetin, garcinone E, psorofebrin
Inflammation	Betulinic acid, mangiferin, psorofebrin, emodin, garcinone E
Oxidative stress	Isocadensin D, gallic acid, mangiferin, psorofebrin, emodin
Skin barrier repair	Psorofebrin, isocadensin D, betulinic acid

**Table 8:** Preclinical toxicity studies supporting the clinical safety profile of *Psorospermum febrifugum.*

**Study method**	**Study population**	**Intervention**	**Controls**	**Outcome**	**Safety aspects**	**Reference(s)**
Cell line toxicity	Human glioblastoma cell lines	Leaves and stem bark extract	Not applicable	No toxic effects observed	Suggests good tolerability	[20]
Acute oral toxicity	Rats	Crude ethanolic stem bark extract (up to 5000 ppm)	Not applicable	No deaths observed	May be safe at high doses	[13, 14, 27]
Acute oral toxicity	Mice	Stem bark extract (up to 5000 mg/kg)	Not applicable	No deaths or clinical signs of toxicity	May be safe at high doses	[8, 13, 14]
Acute dermal toxicity	Rabbits	Aqueous stem bark extract (up to 10,000 mg/kg)	Not applicable	No adverse effects on the skin, liver, and kidneys	May be safe for topical use	[27]

## Data Availability

Data supporting the findings are included in the manuscript tables.
